# Intracellular and in vivo activities of oxazolidinone drugs against *Mycobacterium avium* complex infection

**DOI:** 10.1038/s41598-023-48001-y

**Published:** 2023-11-23

**Authors:** Ju Mi Lee, Lee-Han Kim, Su-Young Kim, Byung Woo Jhun, Wonsik Lee, Sung Jae Shin

**Affiliations:** 1 Department of Microbiology, Institute for Immunology and Immunological Disease, Graduate School of Medical Science, Brain Korea 21 Project, Yonsei University College of Medicine, Seoul, South Korea; 2 Division of Pulmonary and Critical Care Medicine, Department of Medicine, Samsung Medical Center, Sungkyunkwan University School of Medicine, Seoul, South Korea; 3 School of Pharmacy, Sungkyunkwan University, Suwon, South Korea

**Keywords:** Antimicrobials, Bacteria, Cellular microbiology, Clinical microbiology, Pathogens

## Abstract

The prevalence of *Mycobacterium avium* complex-pulmonary disease (MAC-PD) has become a growing concern worldwide, and current treatments involving macrolides (clarithromycin [CLR] or azithromycin), ethambutol, and rifampicin have limited success, highlighting the need for better therapeutic strategies. Recently, oxazolidinone drugs have been identified as novel anti-tuberculosis drugs effective against drug-resistant *M. tuberculosis*. However, the effects of these drugs against MAC are still controversial due to limited data. Here, we first evaluated the intracellular anti-MAC activities of two oxazolidinone drugs, linezolid (LZD) and delpazolid (DZD), against 10 macrolide-susceptible MAC strains and one macrolide-resistant *M. avium* strain in murine bone marrow-derived macrophages (BMDMs) and found that both drugs demonstrated similar potential. The synergistic efficacies with CLR were then determined in a chronic progressive MAC-PD murine model by initiating a 4-week treatment at 8 weeks post-infection. Upon assessment of bacterial burdens and inflamed lesions, oxazolidinone drugs exhibited no anti-MAC effect, and there was no significant difference in the synergistic effect of CLR between LZD and DZD. These findings suggest that oxazolidinone drugs inhibit intracellular bacterial growth, even against macrolide-resistant MAC, but their clinical application requires further consideration.

## Introduction

Nontuberculous mycobacteria (NTM) are recognized as opportunistic infections that can result in chronic pulmonary disease, besides *Mycobacterium tuberculosis* complex and *M. leprae*. The incidence and prevalence of NTM infections have increased worldwide and have surpassed those of *M. tuberculosis* infections in some countries^1,2^. Among the expanding group of NTM, *M. avium* complex (MAC) is considered the main causative agent responsible for this disease, and composed of two species, *M. avium* and *M. intracellulare*^3,4^.

To effectively combat MAC infection, patients diagnosed with MAC disease are required to adhere to a complex therapeutic regimen consisting of at minimum three antibiotics, including a macrolide (such as clarithromycin [CLR] or azithromycin), rifampicin, and ethambutol, for a period of 12–24 months, as recommended in current treatment guidelines^5^. Despite efforts to treat MAC-PD, patients have experienced varying rates of culture conversion, with percentages ranging from 50 to 80%. Additionally, recurrence rates have been reported between 25 and 48%, and reinfection rates ranging from 46 to 75%^6–8^. Thus, treating MAC disease remains a formidable challenge due to the uncertain efficacy of individual antibiotics and the potential for long-term treatment to induce undesirable side effects^9,10^. Presently, the clinical treatment for NTM infection is limited, with the repurposing and reformulation of antibiotics serving as the primary treatment approach^11^. Consequently, the limited therapeutic options and the prolonged duration of treatment for MAC infection highlight the urgency of identifying novel and effective antimicrobial agents.

Linezolid (LZD), a pioneering oxazolidinone antibiotic, is known to exhibit bactericidal activity by binding to domain V of 23S rRNA and curtailing the biosynthesis of bacterial proteins at an early stage of translation in the management of multidrug-resistant tuberculosis (MDR-TB)^12^. LZD has been preferred as one of the first-line components in a multidrug regimen (used with macrolide from the initial phase guided by in vitro drug susceptibility testing [DST]) for *M. abscessus* disease, owing to the potent bactericidal action of oxazolidinone antibiotics^13–15^. However, in vitro and in vivo correlations for MAC-PD treatment have not been verified^14,16,17^. Therefore, the importance of using LZD is still controversial. LZD has been recommended as one of the alternative drugs for MAC-PD patients whose isolate is resistant to first-line drugs^14^. Moreover, Deshpande et al. reported that LZD showed a bactericidal effect (1.0 log10 colony-forming units [CFUs] per mL kill) against MAC following the Monte Carlo simulations to identify the optimal clearance dose of LZD for MAC-PD patients^18^. However, they also recommended caution in its use due to the potential association of the optimal dose, 1800 mg/day, with a high rate of adverse events^18^. Furthermore, LZD is not a suitable option for long-term therapy, as it has been associated with significant adverse effects^16,19–21^.

Several studies have endeavored to explore delpazolid (DZD), an innovative oxazolidinone drug incorporating a cyclic amidrazone moiety, coded as LCB01-0371, as a prospective replacement for LZD, concerning pharmacokinetics, safety, and tolerability against *Mycobacterium* species^22–26^. Through Phase 1 and 2 clinical trials, DZD has shown potential in alleviating myelosuppression and effectively curtailing the duration of TB therapy^11,27^. Notably, Kim et al. have posited that DZD exhibits tremendous potential as a promising therapeutic agent with enhanced safety profiles to supplant LZD in the treatment of *M. abscessus*^21^.

Though various methods, ranging from in vitro DST to clinical trials, have been used to evaluate the activity of DZD in TB and other NTM diseases, in vitro DST has been the sole method used to assess the activity of DZD in MAC disease^21,23,28–32^. Recently, the in vitro effectiveness of oxazolidinone drugs (LZD, DZD, and sutezolid [SZD]) against MAC strains was compared, revealing that DZD and LZD had similar higher minimum inhibitory concentration (MIC) values than SZD against clinical MAC strains, including both macrolide-susceptible and macrolide-resistant strains^28^. Consequently, there is a lack of significant studies specifically demonstrating the intracellular and in vivo anti-MAC activity of oxazolidinones.

Here, we aspire to assess the effectiveness of oxazolidinone drugs as reliable antibiotics to combat MAC-PD by meticulously examining their potential in MAC-infected bone marrow-derived macrophages (BMDMs). Our ultimate objective is to evaluate the potency of oxazolidinone drugs, both individually and in combination with CLR treatment, in a murine model that has been infected with *M. avium*.

## Results

### In vitro DST of oxazolidinone drugs

Before conducting the intracellular anti-MAC activity testing, the in vitro efficacies of oxazolidinone drugs (LZD and DZD) were meticulously evaluated following the Clinical and Laboratory Standards Institute (CLSI) guideline (Table 1)^33^. Consistent with recent in vitro studies that assessed the MIC of clinical macrolide-susceptible MAC strains^28,30^, the clinical strains used in our study exhibited relatively high MIC values for DZD.

**Table 1:** MICs (mg/L) and FICI of CLR and oxazolidinone drugs against *M. avium* complex.

Species	Strains	MIC (single drug)			MIC (combined)		FICI (effect)	
		CLR	LZD	DZD	CLR/LZD	CLR/DZD	CLR/LZD	CLR/DZD
*M. avium*	CP000479.1*	0.5	16	16	0.0625/8	0.125/8	0.625 (A)	0.75 (A)
	ATCC 700898*	1	16	8	0.5/4	0.5/4	0.75 (A)	1 (A)
	SMC #5	2	32	32	0.25/16	0.25/16	0.625 (A)	0.625 (A)
	SMC #6	4	8	8	0.5/4	1/4	0.625 (A)	0.75 (A)
	SMC #7	0.5	4	2	0.0625/2	0.125/1	0.625 (A)	0.75 (A)
*M. intracellulare*	ATCC 13950*	0.25	1	4	0.0625/0.5	0.125/4	0.625 (A)	0.75 (A)
	SMC #7	1	8	32	0.125/4	1/16	0.625 (A)	1.5 (I)
	SMC #8	4	16	64	2/2	2/16	0.625 (A)	0.75 (A)
	SMC #9	0.25	4	16	0.125/4	0.125/4	1.5 (I)	0.75 (A)
	SMC #12	0.25	4	8	0.125/1	0.125/8	0.75 (A)	1(A)

The FICI was interpreted as follows: S, synergy for FICI < 0.5; A, additive for 0.5 ≤ FICI ≤ 1.0; and I, indifference for 1.0 < FICI < 4.0

*MIC* minimum inhibitory concentration, *FICI* fractional inhibitory concentration index, *CLR* clarithromycin, *LZD* linezolid, *DZD* delpazolid, *SMC* samsung medical center.

*These strains were used as reference strains in the relevant experiments of this investigation.

Furthermore, since both macrolide and oxazolidinone drugs target the 50 s ribosomal subunits but have different specific target sites^34^, we investigated the fractional inhibitory concentration index (FICI) values of the drug combinations to assess the potential synergistic effect between macrolide and oxazolidinone drugs against MAC strains. These oxazolidinone drugs showed additive results with macrolide for most of the tested MAC strains (Table 1).

### Intracellular anti-MAC activity of oxazolidinone drugs

**Figure 1:**
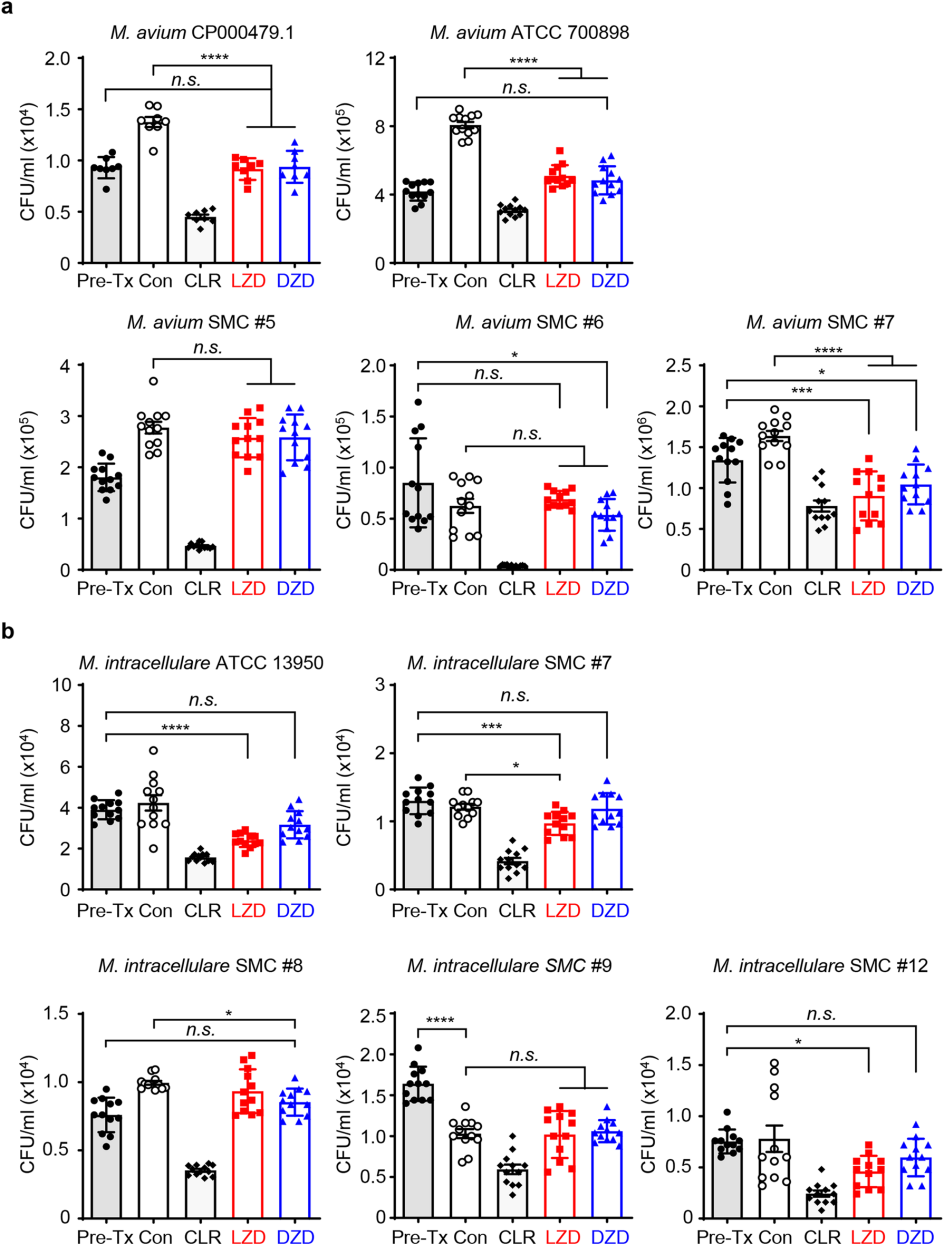
The comparison of the intracellular activities of oxazolidinone drugs against a variety of MAC strains in BMDMs. The BMDMs were infected with different MAC strains, including (**a**) *M. avium* type strains (*M. avium* CP000479.1, *M. avium* ATCC 700,898), *M. avium* clinical strains (*M. avium* SMC #5, *M. avium* SMC #6, and *M. avium* SMC #7), (**b**) *M. intracellulare* type strain (*M. intracellulare* ATCC 13,950) and *M. intracellulare* clinical strains (*M. intracellulare* SMC #7, *M. intracellulare* SMC #8, *M. intracellulare* SMC #9, and *M. intracellulare* SMC #12). The cells were treated with 10 mg/L of indicated drugs, and the bacterial CFUs were measured at 3 days post-infection. The experiments were repeated at least twice independently, and the results of a representative experiment are presented. Each dot on the graph represents the mean value ± S.D. of duplicate or triplicate wells, with four spots applied per well. The statical analysis was performed using the non-parametric Mann–Whitney *U* test and represented as the mean value ± S.D. **p < 0.01, ***p < 0.001, ****p < 0.0001. pre-Tx, the day of infection (before treatment); Con, 3 days after infection and no drugs; *CLR* clarithromycin, *LZD*, linezolid, *DZD* delpazolid.

To further examine the protective properties of the two oxazolidinone drugs against intracellular MAC, we assessed their anti-MAC efficacy, specifically their capacity to inhibit intracellular MAC growth, and their potential to serve as a substitute for CLR. As the first step, BMDMs were infected with five different strains of *M. avium* and five strains of *M. intracellulare*, respectively. To compare their intracellular activity at an equivalent concentration to CLR (the positive control antibiotic), the infected cells were treated with 10 mg/L of CLR, a concentration that had previously demonstrated efficacy^35^. The same concentration, 10 mg/L, was equally applied to oxazolidinone drugs. The cells were then exposed to this treatment for 3 days to observe their intracellular response. In Fig. 1, all bars are labelled as follows: ‘pre-Tx’, representing the time point before treatment, 4 h post-infection control; ‘Con’, representing 3 days post-infection and untreated control; ‘CLR’, representing clarithromycin treatment; ‘LZD’, representing linezolid treatment; ‘DZD’, representing delpazolid treatment. If the bacterial growth is not significant after oxazolidinone drug treatment compared to ‘pre-Tx’, but it significantly and slightly grows compared to ‘Con’, it indicates a bacteriostatic effect. If the bacterial growth significantly decreases after oxazolidinone drug treatment compared to ‘pre-Tx’, it indicates a bactericidal effect. As a result, the oxazolidinone drugs showed beneficial growth-inhibitory effects against three out of five *M. avium* strains, compared with ‘pre-Tx’ and ‘Con’, although they did not achieve the same level of efficacy as CLR. Not surprisingly, given the higher MIC value of DZD compared to LZD against *M. intracellulare* strains, LZD, not DZD, exhibited statistically significant anti-MAC activity in three out of five *M. intracellulare* strains (Fig. 1). Moreover, the similar anti-mycobacterial effect of both oxazolidinone drugs on intracellular *M. tuberculosis* H37Rv was exhibited at the same concentration (Supplementary Fig. 1). Collectively, when treated with a lower concentration than the MIC value, oxazolidinone drugs are sufficient to inhibit intracellular bacterial growth, but the degree of efficacy may vary.

To determine whether these effects were due to concentrations lower than the MIC values, we exposed BMDMs to various concentrations of LZD and DZD ranging from 6.25 to 100 mg/L for 24 h and evaluated the effect on cell viability (Supplementary Fig. 2A). These high oxazolidinone concentrations did not affect cell viability, and a statistically significant difference was only found between the untreated control and CLR concentrations of ≥ 25 mg/L. Subsequently, BMDM were infected with two strains with relatively low MIC values, *M. avium* SMC #7 and *M. intracellulare* ATCC 13950, and were exposed to the highest concentration (100 mg/L) for 3 days. Interestingly, both LZD and DZD strongly inhibited intracellular bacterial growth (Supplementary Fig. 2B and C). Based on our findings, it appears that the oxazolidinone drugs at the concentration of 100 mg/L can produce an equivalent effect to CLR at a concentration of 10 mg/L against intracellular bacteria of *M. avium* SMC #7. However, for *M. intracellulare* ATCC 13950, these drugs did not show the same effect as CLR. In addition, we investigated whether oxazolidinone drugs could effectively inhibit the growth of macrolide-resistant *M. avium* SMC #422. In vitro DST conducted against *M. avium* SMC #422 revealed that LZD and DZD demonstrated inhibitory effects on bacterial growth at concentrations of 4 mg/L and 8 mg/L, respectively (Supplementary Fig. 3A). Moreover, we evaluated the intracellular anti-MAC activity of oxazolidinone drugs at both lower (10 mg/L) and higher (100 mg/L) concentrations. Interestingly, both oxazolidinone drugs exhibited activity against *M. avium* SMC #422, effectively restraining its growth within macrophages, but notably at 100 mg/L rather than 10 mg/L (Supplementary Fig. 3B). Taken together, these findings indicate that the effectiveness of low concentrations of oxazolidinone drugs in inhibiting intracellular MAC growth can vary depending on the genetic diversity of MAC strains. Additionally, the metabolic and immune responses of the infected host cell may be influenced by the diversity of MAC strains, potentially impacting the efficacy of the drug.

### Evaluation of oxazolidinone drugs in combination with CLR in a chronic *M*. *avium*-pulmonary infection murine model

**Figure 2:**
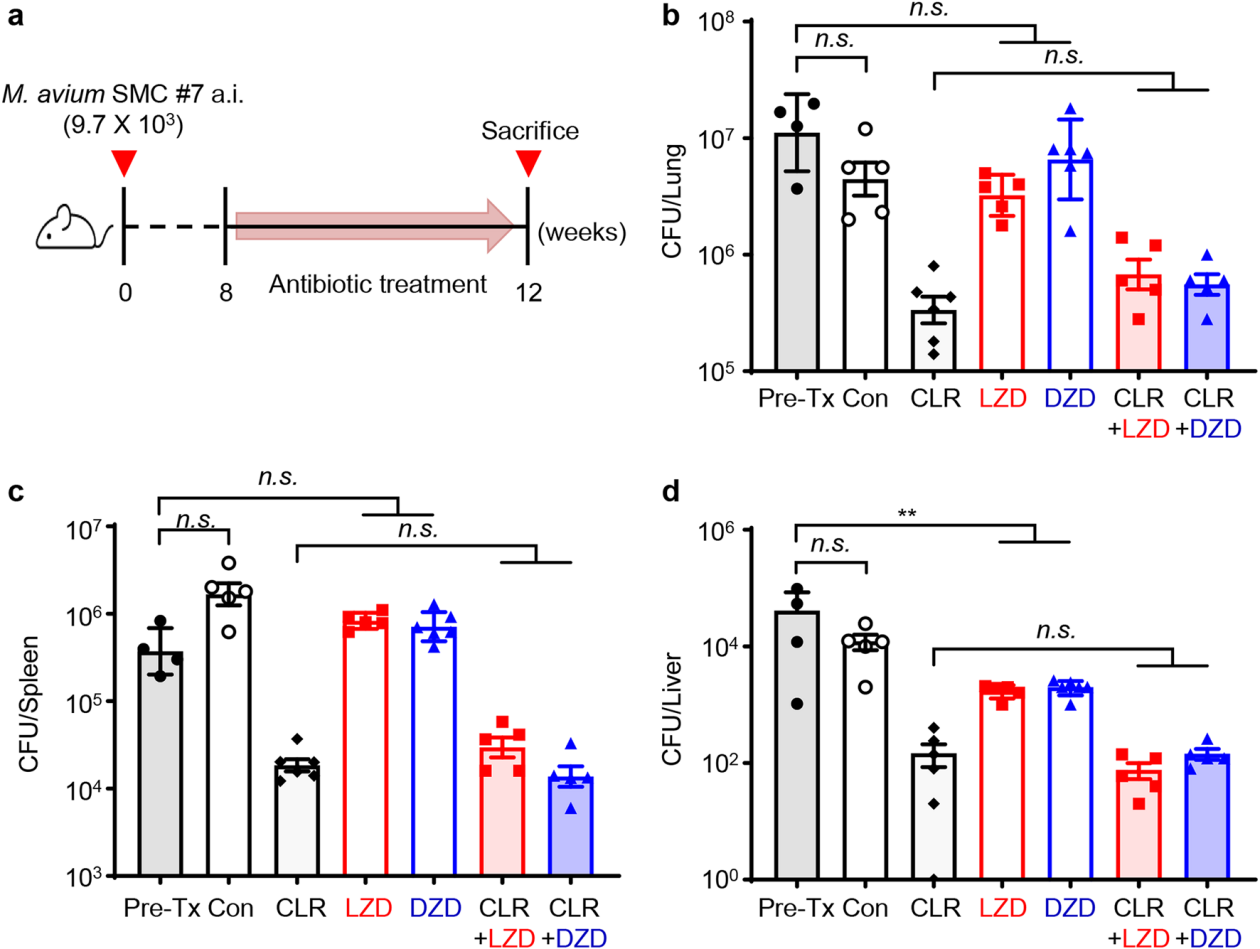
Comparative assessment of the synergistic effects of LZD and DZD with CLR in a chronic progressive murine model of *M. avium*-PI. (**a**) Schematic design for the in vivo experiment. At eight weeks after BALB/c mice were infected with *M. avium* SMC #7 via aerosolization, four weeks of daily oral treatment was initiated with oxazolidinone drugs based on CLR therapy. The daily dose for all drugs was 200 mg/kg. After 4 weeks of treatment, the mice were euthanized, and the lungs, spleens, and livers were assessed for bacterial counts and histopathological examination. The results are represented in bar graphs representing mean CFU counts in the (**b**) lungs, (**c**) spleens and (**d**) livers. The statistical significance in (**b**–**d**) was calculated by the non-parametric Mann–Whitney *U* test, and the results are shown as the mean value ± S.D. *p < 0.05, **p < 0.01 and *n.s*. not significant. *A*.*i*. aerosol infection; pre-Tx, 8 weeks post-infection (before treatment); Con, 12 weeks post-infection (after treatment); *CLR* clarithromycin, *LZD* linezolid, *DZD* delpazolid, *CLR* + *LZD* clarithromycin with linezolid, *CLR* + *DZD* clarithromycin with delpazolid.

To assess the in vivo synergistic efficacy of oxazolidinone in combination with CLR, we established a chronic progressive BALB/c model of *M. avium*-pulmonary infection (PI) through aerosol infection with *M. avium* SMC #7. This strain was chosen due to its demonstrated high sensitivity in both MIC and intracellular activity evaluations with two oxazolidinones (Table 1, Fig. 1 and Supplementary Fig. 2). Furthermore, it had been previously confirmed to induce significant lung lesions in a MAC-pulmonary disease model in BALB/c mice. This makes it a suitable choice for validating treatment regimens for MAC infections^35^. Subsequently, at eight weeks after infection, we administered each drug as monotherapy or in combination at a dose of 200 mpk for four weeks, as illustrated in Fig. 2a. The dosages of CLR and LZD were determined based on previous studies^21,36–38^. Moreover, DZD was administered at the same dosage as LZD because it has been reported to have similar in vivo pharmacokinetic profiles to LZD, with no observed adverse effects at doses up to 1200 mg/day^21^. Therefore, we opted for the same doses for LZD and DZD. The groups of infected groups were categorized as follows: pre-Tx, pre-treatment group sacrificed at 8 weeks post-infection; Con, untreated infection control group sacrificed at 12 weeks post-infection; CLR, clarithromycin-treated group; LZD, linezolid treatment group; DZD, delpazolid treatment group; CLR + LZD, clarithromycin and linezolid-treated group; CLR + DZD, clarithromycin and delpazolid-treated group.

First, the mice exhibited a diminished capacity to regulate the *M. avium* SMC #7 infection, leading to notably elevated bacterial counts in lung, spleen, and liver tissues at distinct time points-specifically at 0, 8, and 12 weeks post-infection, as illustrated in Supplementary Fig. 4. Analysing the growth pattern, there was an accelerated increase in bacterial burden from 0 to 8 weeks, followed by a relatively stable phase from 8 to 12 weeks in the lungs, corresponding with the levels of pro- and anti-inflammatory cytokines (Supplementary Fig. 5). Our data correlates with previous studies that reported a relationship between bacterial growth patterns and immune responses^39–41^. Subsequently, we assessed the efficacy of the intervention by quantifying the mean CFU to determine its impact on the bacterial burden (Fig. 2b–d). As a result, oxazolidinone monotherapy did not show significant anti-MAC activity based on ‘pre-Tx’ and ‘Con’ groups in the lung and spleen. In the liver, both oxazolidinone drugs showed significant anti-MAC activity (*p* < 0.01). However, there were no statistically significant differences between CLR combined with LZD or DZD treatment and CLR monotherapy. These results suggest that there was no improvement in the anti-MAC activity of CLR in murine model.

**Figure 3:**
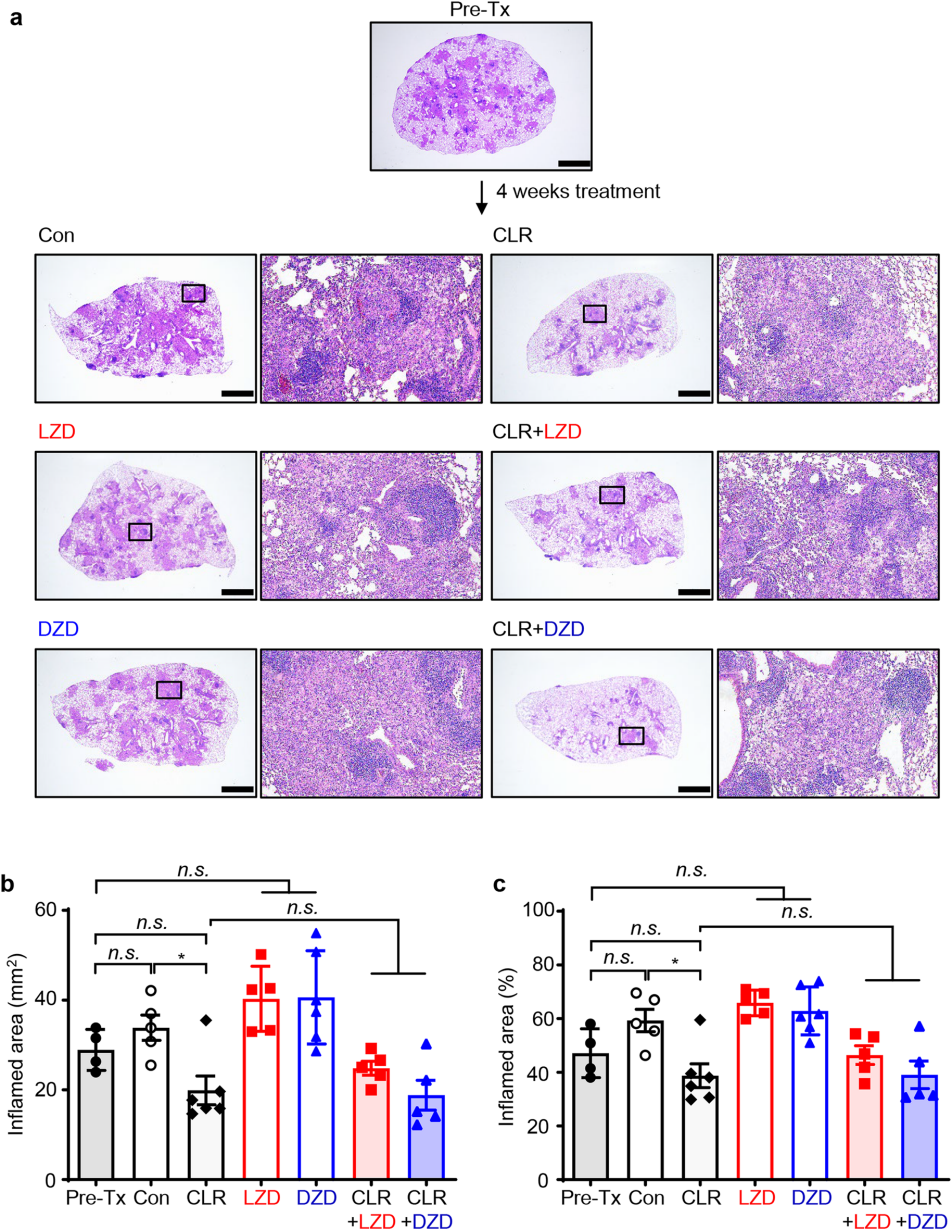
Histopathological assessment of lung inflammation in mice treated with LZD and DZD in combination with CLR at four weeks post-treatment. The panel (**a**) displays the 10 × magnification photomicrographs of the right superior lung lobe stained with H&E (scale bars = 2 mm), and the upper part of each image indicates the respective treatment group. The bar graphs in (**b**) and (**c**) illustrate the quantification of the size and the percentage of H&E-stained inflamed areas, respectively. The statistical significance of the results in (**b**) and (**c**) was analyzed by the non-parametric Mann–Whitney *U* test, and the mean value ± S.D. is presented in the graphs. *p < 0.05 and *n.s*. not significant. pre-Tx, 8 weeks post-infection (before treatment); Con, 12 weeks post-infection (after treatment); *CLR* clarithromycin, *LZD* linezolid, *DZD* delpazolid, *CLR* + *LZD* clarithromycin with linezolid, *CLR* + *DZD* clarithromycin with delpazolid.

We further investigated whether oxazolidinone drugs have the potential to induce smaller granulomas compared to the ‘pre-Tx’, ‘Con’ and ‘CLR’ groups, as depicted in Fig. 3a. Microscopically, bacterial burdens resulted in small, non-necrotic granulomatous inflammation that occupied over 45% of the lung tissue at 8 weeks, followed by a relatively stable phase from 8 to 12 weeks (from ‘pre-Tx’ to ‘Con’) (Fig. 3b and c). However, no significant differences were found when comparing the groups treated with either of the two oxazolidinone drugs versus ‘Pre-Tx’ and ‘Con’. Moreover, these finding suggest that there was no synergetic effect between CLR and oxazolidinone drugs in the chronic progressive *M. avium*-PI model.

## Discussion

While in vitro DST for MAC is commonly carried out as part of routine practice, the evaluation of intracellular activity with new drugs is rarely conducted. Previously, we established that MICs could not fully reflect all pharmacological activities and that therapeutic efficacy against MAC strains should consider host-mediated control of drug effectiveness using intracellular and in vivo infection models^35^. In this study, we evaluated the anti-MAC activity of oxazolidinone drugs against MAC strains, including both macrolide-susceptible and macrolide-resistant strains, for the first time. This evaluation was conducted through in vitro minimum inhibitory concentration (MIC) assays, intracellular assays, and the chronic *M. avium*-PI murine model.

Oxazolidinone drugs are commonly used to inhibit the growth of a wide range of multidrug-resistant gram-positive bacteria^42^. However, in vitro DST has shown that LZD exhibits weak activity against MAC, with MIC values ranging from 16 to 64 mg/L, despite its superior efficacy against various gram-positive pathogenic bacteria and MDR-TB^11,43^. Nevertheless, LZD has been used for a refractory disseminated MAC-infected patient^44^, but the British Thoracic Society guideline disapproves of its use due to the risk of toxicity when combined with CLR, which increases LZD serum levels^45^. Therefore, recent findings have indicated that DZD, among the oxazolidinone drugs, has improved stability and safety through various verifications to overcome the limitations of LZD^24,25,46^. Wang et al. demonstrated through in vitro DST that DZD exhibited more potent activity than LZD against *M. tuberculosis* clinical isolates, including MDR-TB^47^. Furthermore, Cho and Jang demonstrated that both DZD and LZD effectively inhibited *M. tuberculosis* growth in BMDMs^27^. To date, only one in vivo DST has been reported in C57BL/6 infected with NTM. Kim et al*.* evaluated the activity of DZD in cellular and in vivo settings, comparing it to LZD and CLR against *M. abscessus*^21^. Unlike the intracellular activity in our study, DZD at a concentration of 10 mg/L exhibited the ability to reduce *M. abscessus* by 79% in BMDMs. In acute infections with *M. abscessus* in C57BL/6, oxazolidinone drugs at a dose of 100 mpk demonstrated better efficacy compared to CLR at a dose of 200 mpk. Moreover, LZD and DZD showed a very similar effect in *M. abscessus* infection, despite demonstrating statistically significant effects compared to the untreated infection control.

Indeed, several studies have highlighted distinct susceptibility levels to MAC infection across various mouse strains^39,40,48^. Notably, BALB/c mice were recommended as the optimal choice for conducting DST targeting MAC^49^. Furthermore, in our preceding research, we explored the pathogenesis in two distinct mouse strains, BALB/c and C57BL/6, through aerosolization with *M. avium* SMC #7^50^. This investigation revealed that BALB/c mice have a relatively high susceptibility to bacterial infections. Hence, it is plausible that the effectiveness of oxazolidinone drugs may not be dose-dependent, but rather influenced by mouse strains or specific bacterial species.

Briefly, we have confirmed that oxazolidinone drugs effectively inhibit intracellular macrolide-susceptible and macrolide-resistant MAC strains (*M. intracellulare* was more tolerant to DZD than *M. avium*) and *M. tuberculosis* H37Rv in BMDMs. However, there was no noticeable difference between LZD and DZD. In the chronic *M. avium*-PI murine model, oxazolidinone drug monotherapy did not significantly reduce bacterial loads or pulmonary inflammation. Furthermore, neither of the two oxazolidinone drugs exhibited a synergistic effect with CLR in reducing bacterial load or inflamed areas. In general, LZD has been suggested to use for other extrapulmonary NTM diseases, especially *M. abscessus* and *M. chelonae* infection^51^. In our study, the oxazolidinone drugs were slightly effective in reducing bacterial loads in the liver, indicating their potential to inhibit bacterial dissemination. These findings provide valuable in vitro and in vivo evidence to the existing knowledge on the pharmacological efficacy for MAC-PD patients, filling the gap in comparative data on the activity of oxazolidinone drugs.

There are several limitations to consider. Firstly, it may not be sufficient to explain the anti-MAC activity of oxazolidinone drugs based on small populations of MAC strains (n = 10) in vitro DST. Previous studies by Yu et al. and Kim et al. have shown similar MIC distribution patterns and values for LZD and DZD against larger populations of clinical MAC strains (n = 48 and n = 97, respectively)^28,30^. It would be preferable to examine a greater variety of strains, as conducted in these studies. Nevertheless, our results align with those of previous studies^28,30^. Secondly, the observed differences in the growth control ability of 10 MAC strains, when exposed to lower concentrations of oxazolidinone drugs during intracellular activity assessment, are influenced by multiple factors that contribute to the efficacy of these drugs. Factors such as drug penetration, accumulation, bioavailability within BMDMs, and the responsiveness of bacteria to the pharmacological action play a role. The superior growth inhibition ability of *M. avium* compared to *M. intracellulare* may be influenced by differences in the regulation of complex immune and metabolic responses within host cells depending on the bacterial species, potentially affecting the efficacy of the drug. Thirdly, although oxazolidinone drugs demonstrated bacterial growth inhibitory effects in MAC-infected cells, the lack of effectiveness in vivo could be attributed to the variations in immune status between BMDMs and murine models. Considering factors such as drug concentration, infection status, treatment duration, and treatment frequency is crucial when assessing changes in the protective efficacy of drugs in in vivo studies. Further investigation is warranted to explore this aspect in more detail.

In conclusion, our study explored the potential of oxazolidinone drugs as anti-MAC therapy for chronic progressive MAC-PI in the murine model. We evaluated their intracellular activities against various MAC strains in BMDMs, providing insights into their anti-MAC effects. It is important to carefully consider the necessity of using oxazolidinone drugs for treatment based on these findings. Additionally, we observed that oxazolidinone drugs were effective in inhibiting the growth of macrolide-resistant strains, suggesting the potential use of other drugs targeting bacterial protein synthesis in the treatment of macrolide-resistant MAC-PD. These finding highlight the need for further investigation into drug combinations for the treatment of macrolide-resistant MAC-PD.

## Materials and methods

### Ethics statement

The animal experiments were conducted in accordance with the guidelines established by the Korean Food and Drug Administration. The protocols and procedures were approved by the Ethics Committee and Institutional Animal Care and Use Committee at Yonsei University College of Medicine, with permit numbers 2018-0229.

### Mice

Female BALB/c mice aged 6 to 8 weeks, which were specific-pathogen-free, were obtained from Japan SLC, Inc. in Shijuoka, Japan. These mice were provided with a sterile commercial diet and water ad libitum, and were housed in an animal biosafety level-3 facility at a constant temperature of 24 ± 1 °C and humidity of 50 ± 5%, with a 12-h light–dark cycle system. The mice were allowed to adapt for one week before using in any experiments. No visible symptoms were observed in any of the mice during daily monitoring, and none were sacrificed before the endpoint of the experiment.

### Mycobacterial strain and cultivation

A total of 11 clinical strains from the MAC-PD patients were included in this study, comprising six strains of *M. avium* (consisting of five macrolide-susceptible strains and one strain with an acquired-macrolide-resistant phenotype, confirmed to possess a 23S rRNA gene mutation associated with the development of macrolide resistance) and five strains of *M. intracellulare*. Additionally, *M. tuberculosis* H37Rv, was also utilized. In brief, reference strains (*M. avium* CP000479.1, *M. avium* ATCC 700,898, *M. intracellulare* ATCC 13,950, and *M. tuberculosis* H37Rv [ATCC 27294]) were procured from the American Type Culture Collection (ATCC; Manassas, VA, USA), and all clinical strains that were isolated from typical MAC-PD patients at the Samsung Medical Center (SMC; Seoul, South Korea) were used. To prepare single-cell suspensions of each strain, a selected single colony from each Middlebrook 7H10 agar plate (BD-Difco, Le Pont de Claix, France) was cultivated in Middlebrook 7H9 liquid broth (BD-Difco) supplemented with 10% (vol/vol) oleic acid-albumin-dextrose-catalase (OADC) sub-culturing every week during the month at 37 °C with shaking at 145 rpm. Detergent was deliberately avoided during cultivation to prevent any interference. Previous studies have represented that the use of Tween 80 influences the biochemical and phenotypic characteristics of mycobacteria by acting directly on the cell wall^52–55^. Then, bacteria were collected, aliquoted, and stored at -80 °C until use. For infection experiments in macrophages and mice, the number of CFUs was assessed by plating serially diluted bacteria on Middlebrook 7H10 agar plates, as previously described^50^.

### Antibiotics

The following drugs were used in this study: CLR (purchased from Tokyo Chemical Industry, Tokyo), LZD, and DZD (LCB01-0371; provided by LegoChem Biosciences, Inc.). For in vitro and intracellular DST, all drugs were dissolved in dimethyl sulfoxide and diluted in Dulbecco’s phosphate-buffered saline (DPBS; Biowest, Nuaillé, France). For in vivo oral treatment, the drugs were dissolved in 0.5% carboxymethylcellulose.

### Minimum inhibitory concentration

As previously described^35^, the in vitro DST for CLR, LZD, and DZD was performed using the broth microdilution resazurin assay after 7 days of incubation. The MIC of the antibiotics was determined according to the CLSI guidelines^33^. To confirm the MIC results for the tested MAC strains, the test was repeated at least twice (in duplicate wells).

### Checkerboard test of oxazolidinone drugs and macrolide combinations

Checkerboard assay was employed using MAC strains to evaluate the synergistic activity of CLR in combination with either LZD or DZD, following the methodology described in previous studies^56–58^. In this assay, a total of 100 μl of the bacterial suspension was added to 96-well microplates, as per established protocols. A twofold serial dilution of LZD and DZD, respectively, ranging from 128 to 0.5 mg/L, was added to each row, while CLR, diluted from 4 to 0.0625 mg/L, was added to each column. Each drug combination was tested in duplicate for statistical validity. The microplates were then incubated at 37 °C for 7 days, and subsequent bacterial growth was visually assessed. To determine the interaction between the drugs, the FICI was calculated using the formula FIC = (MIC_a_ combination/MIC_a_ alone) + (MIC_b_ combination/MIC_b_ alone), where “a” and “b” represent CLR and the oxazolidinone drugs, respectively. The FICI values were interpreted as follows: FICI ≤ 0.5, synergy; 0.5 < FICI ≤ 1.0, an additive effect; 1.0 < FICI < 2.0, indifference; FICI < 2.0, antagonism.

### Intracellular anti-MAC activity of individual drugs

BALB/c BMDMs were differentiated in Dulbecco’s modified Eagle’s medium (DMEM; Biowest) supplemented with 10% heat-inactivated fetal bovine serum (FBS; Biowest), 10% L929 supernatant and 1% penicillin/streptomycin (P/S). The intracellular anti-MAC activity of the indicated antibiotics was then tested, as previously described^35^. Briefly, BMDMs (3 × 10^5^ cells/ml) were cultured in a 48-well plate for a day. Then, to exclude any potential effects of P/S and to ensure optimal infection conditions, we conducted infection experiments in BMDMs using DMEM containing 5% FBS without the addition of P/S. This approach aimed to provide a suitable environment for infection and minimize any confounding factors associated with the presence of P/S. Afterward, frozen stocks of mycobacteria (both macrolide-susceptible and macrolide-resistant MAC strains, as well as with *M. tuberculosis* H37Rv) were thawed, and BMDMs were infected. The infections were carried out at a multiplicity of infection (MOI) of 3 for the MAC strains and an MOI of 1 for *M. tuberculosis* H37Rv for 4 h. The cells were then washed with DPBS to remove extracellular bacteria and incubated in new DMEM containing 5% FBS with or without drugs in duplicate or triplicate wells for 3 days. After that, the cells were lysed with 0.05% Triton X-100, and the lysates were serially diluted in DPBS and spotted four times per well on 7H10-OADC agar plates to determine the number of viable bacterial CFUs. The CFUs were counted after at least two weeks of incubation with 5% CO_2_ at 37 °C, and the results are expressed as mean CFU values ± standard deviation (S.D) per ml of BMDMs. All representative data were independently repeated at least twice.

### In vivo efficacy of drug combinations in a chronic *M*. *avium*-PI murine model

The efficacy of oxazolidinone drugs administered alone versus in combination with CLR was contrasted in a chronic *M. avium*-PI murine model involving a total of 36 BALB/c mice infected with *M. avium* SMC #7 (2.9 × 10^4^ per mouse) through aerosolization using an inhalation exposure system (Glas-Col in Terre Haute, Indiana, USA), as documented in previous studies^35,59^. For infection, a frozen stock of *M. avium* SMC #7 was thawed and cultured in 7H9 medium with 10% OADC for seven days to enhance bacterial numbers. Subsequently, mice were infected via aerosolization. During the establishment phase of the infection, four mice were euthanized at 8 weeks. At precisely 8 weeks post-infection, mice were orally administrated 200 mpk of CLR, LZD, and DZD, delivering 200 μl per mouse and per day through gavage for 4 weeks, 5 days a week. The drug powders for in vivo oral treatment were suspended in 0.5% carboxymethylcellulose (CMC solution; Sigma-Aldrich, St. Louis, MO, USA). Additionally, combined therapies containing CLR and LZD, or CLR and DZD, were mixed in 0.5% CMC solution, respectively. Control mice received oral administration of 0.5% CMC solution. Lastly, a test group of five to six mice per group was euthanized at 12 weeks post-infection to assess the bacterial load in the lung, spleen, and liver, and conduct a histopathological evaluation of the lung. At both 8 and 12 weeks after the challenge, the mice were humanely euthanized using CO_2_ followed by cervical dislocation, and their tissues were subsequently collected for analysis.

Following tissue harvesting, homogenates of the left lobe of the lungs, the whole spleen, and the left lobe of the livers were plated via serial dilution onto 7H10 agar plates enriched with 10% OADC and 0.5% amphotericin B (Sigma-Aldrich), and the bacterial colonies were counted after incubation under 5% CO_2_ at 37 °C for 2 weeks. Then, for histopathological analysis, the fixed right superior lung lobe was embedded in paraffin and sectioned into four- to five-micrometer sections for evaluation of lung inflammation using hematoxylin and eosin (H&E) staining. The inflamed area of the lung was analyzed using open-source ImageJ software (the National Institutes of Health, Bethesda, MD, USA), and presented as both percentage values and in square millimeters (mm^2^).

### Statistical analysis

The non-parametric Mann–Whitney *U* test from GraphPad Prism version 7.00 (GraphPad Software, La Jolla, CA, USA, www.graphpad.com) was used to determine the statistical significance of the difference between groups, respectively. The results are presented as mean value ± S.D. A significance level of *p* < 0.05 was employed.

### Adherence to ARRIVE guidelines

In all instances involving animals, we strictly followed the ARRIVE guidelines 2.0 (https://arriveguidelines.org/arrive-guidelines), ensuring that each specific experimental approach adhered to the essential 10 and recommended set of details.

### Supplementary Information

Supplementary Information.

## Data Availability

All datasets presented in the study are included in the article/Supplementary Material.
