# Supplementary material for: Intracellular and in vivo activities of oxazolidinone drugs against Mycobacterium avium complex infection
Source: Sci Rep. 2023 Nov 23;13:20631. doi: 10.1038/s41598-023-48001-y (PMC10667338; doi:10.1038/s41598-023-48001-y)
Supplement: Supplementary file 1 — Supplementary Information. [file 41598_2023_48001_MOESM1_ESM.docx]

**Supplemental information**


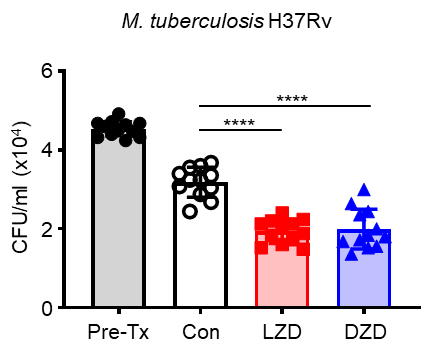


**Figure S1.** Comparative assessment of the intracellular activities of oxazolidinone drugs against *M. tuberculosis* H37Rv in BMDMs. BMDMs were infected with *M. tuberculosis* H37Rv, and then treated with indicated drugs at a concentration of 10 mg/L. The bacterial CFU were measured at 3 days post-infection. The experiments were repeated at least twice independently, and the results of a representative experiment are presented. Each dot on the graph represents the mean value ± S.D. of duplicate or triplicate wells, with four spots applied per well. The statical analysis was performed using the non-parametric Mann-Whitney *U* test and represented as the mean value ± S.D. *****p* < 0.0001. pre-Tx, the day of infection (before treatment); Con, 3 days after infection and no drugs; LZD, linezolid; DZD, delpazolid.


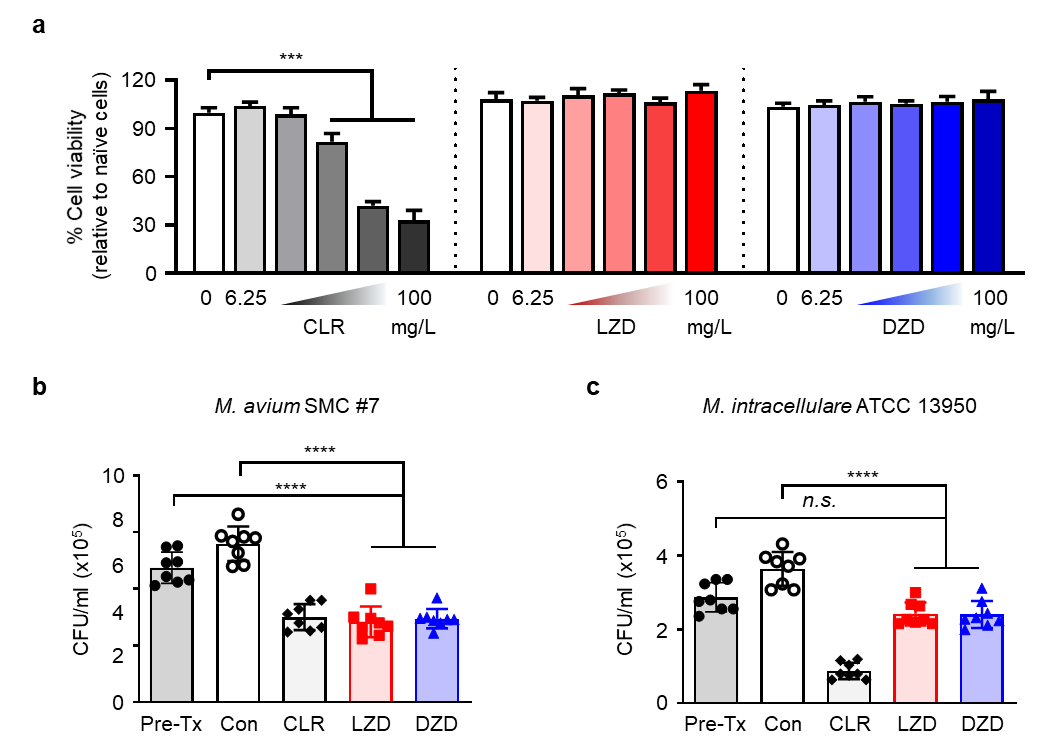


**Figure S2.** Comparative evaluation of the intracellular activities of high dose of oxazolidinone drugs against two MAC strains in BMDMs. (A) The cytotoxicity of the drugs on BMDMs was analyzed after 24 h treatment. Next, BMDMs were infected with (B) *M. avium* SMC #7 and (C) *M. intracellulare* ATCC 13950, treated with 100 mg/L of oxazolidinone drugs and 10 mg/L CLR for 3 days, and the bacterial CFUs were assessed at 3 days post-infection. The experiments were repeated at least twice independently, and the results of a representative experiment are presented. Each dot on the graph represents the mean value ± S.D. of duplicate or triplicate wells, with four spots applied per well. The statical analysis was performed using the non-parametric Mann-Whitney *U* test and represented as the mean value ± S.D. ****p* < 0.001 and *****p* < 0.0001. pre-Tx, the day of infection (before treatment); Con, 3 days after infection and no drugs; Con, untreated infection control; CLR, clarithromycin; LZD, linezolid; DZD, delpazolid.


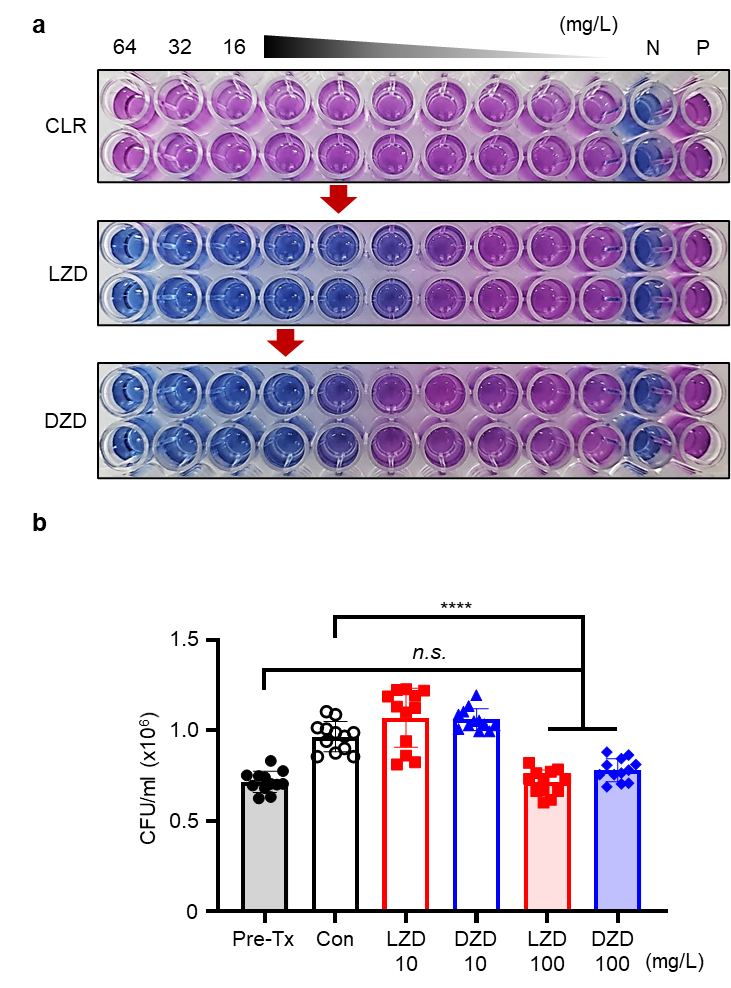


**Figure S3.** Comparative evaluation of *in vitro* and intracellular activities of oxazolidinone drugs against a macrolide-resistant strain of MAC (*M. avium* SMC #422). (A) The MICs of the indicated drugs were determined using the resazurin assay. All drugs were tested across a range of concentrations, starting from 64 mg/L and serially diluted. CLR was utilized to confirm macrolide resistance, and the MIC values of the drugs are highlighted by a red arrow. (B) BMDMs were infected with *M. avium* SMC #422 and subsequently treated with oxazolidinone drugs at concentrations of 10 and 100 mg/L for a duration of 3 days. The bacterial CFUs were assessed at 3 days post-infection. The experiments were repeated independently at least twice, and the results of a representative experiment are presented. Each dot on the graph represents the mean value ± S.D. of triplicate wells, with four spots applied per well. The statical analysis was performed using the non-parametric Mann-Whitney *U* test and represented as the mean value ± S.D. *****p* < 0.0001. CLR, clarithromycin; LZD, linezolid; DZD, delpazolid; N, negative control (7H9-OADC broth only); P, positive control (Bacteria in 7H9-OADC broth without drugs); pre-Tx, the day of infection (before treatment); Con, 3 days after infection and no drugs; CLR, clarithromycin; LZD, linezolid; DZD, delpazolid.

**
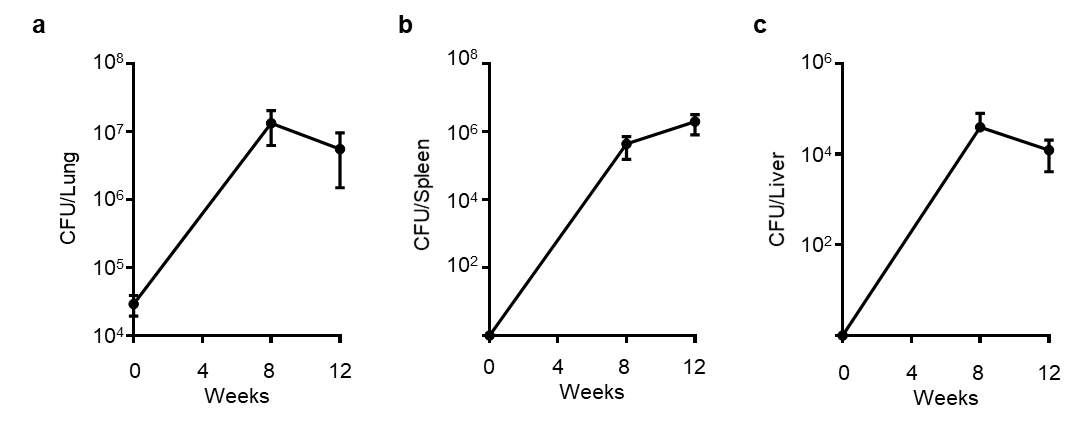
**

**Figure S4.** Bacterial growth kinetics in a chronic progressive murine model of *M. avium*-PI. The mice were euthanized at 0, 8, and 12 weeks after *M. avium* SMC #7 infection, and the results are presented in line graphs with dot plots illustrating the mean CFU counts in the (A) lungs, (B) spleens, and (C) livers.

**
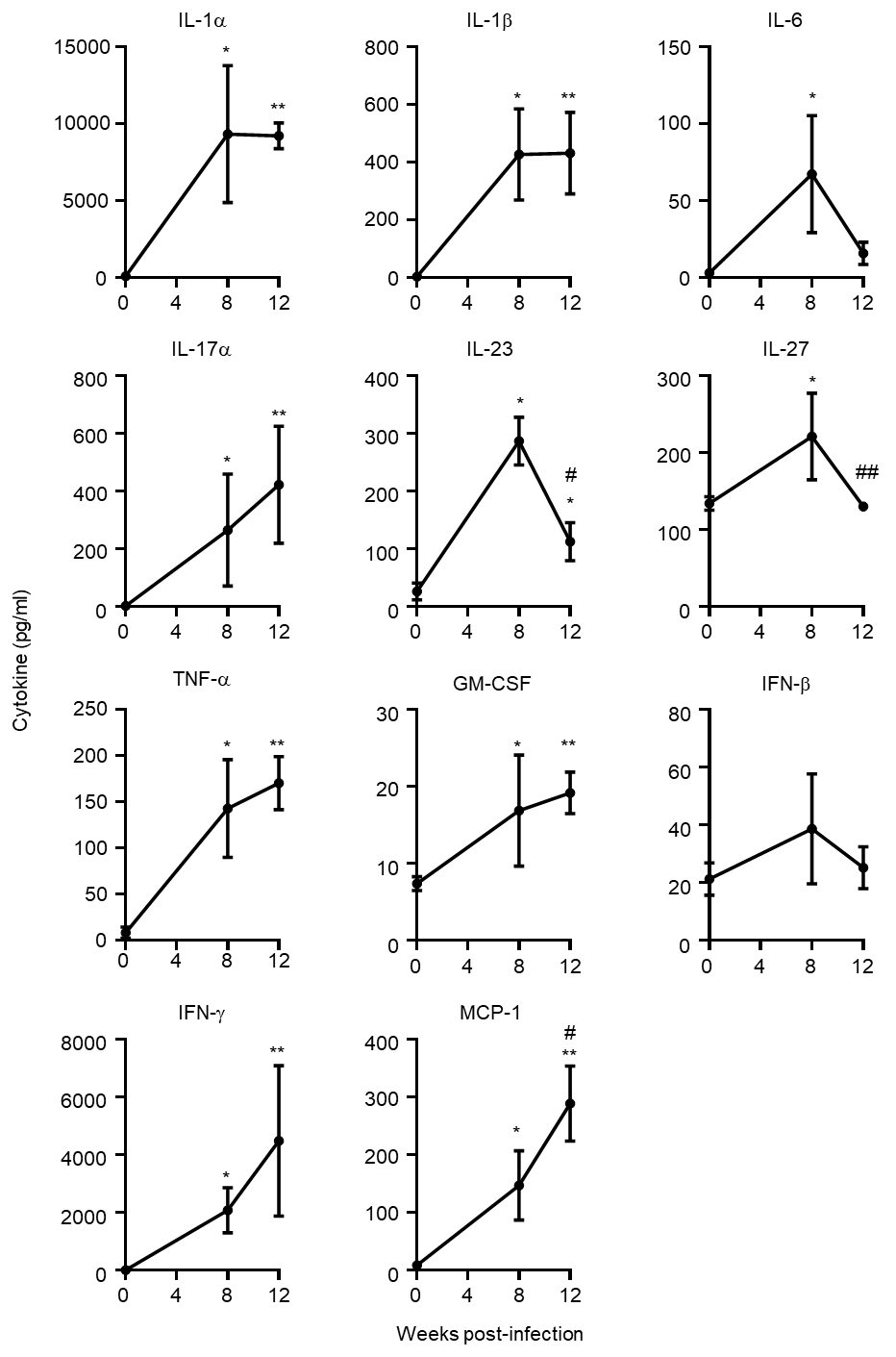
**

**Figure S5.** Time-dependent cytokine profiles of lung lysates of mice in a chronic progressive murine model of *M. avium*-PI. Secreted cytokines in the collected supernatants of lung lysates were quantified by a multiple cytokine array, and the results as presented as line graphs with dot plots. The statical analysis was performed using the non-parametric Mann-Whitney *U* test and represented as the mean value ± S.D. The asterisks represent significant differences between groups 0 weeks vs. 8 weeks or 0 weeks vs. 12 weeks, **p* < 0.05 and ***p* < 0.01. The crosshatches represent significant differences between groups 8 weeks vs. 12 weeks, #*p* < 0.05 and ##*p* < 0.01.

**Supplemental Methods**

**Cell cytotoxicity test**

BMDMs were seeded at a density of 5 × 10^4^ cells/well in a 96-well microplate. Subsequently, the indicated drugs, respectively, were treated to each well, initiating a stepwise dilution series starting from the highest concentration of 100 mg/L, and cascading down to a minimum concentration of 6.25 mg/L. To assess cell viability, the CCK-8 cell cytotoxicity test kit agent (Dojindo Laboratories, Kumamoto, Japan) was added to the wells. The plate was then incubated under optimal conditions of 37°C and 5% CO_2_ for 1 h. Measurement and analysis of the samples were carried out using an Epoch microplate spectrophotometer (BioTek Instruments, VT, USA), with absorbance readings recorded at 450 nm.

**Cytokine quantification using a multiplex cytokine array**

Time-dependent cytokine concentrations in *M. avium*-infected mice were measured using a commercial kit according to the manufacturers’ protocol. Supernatants collected from harvested lung homogenates were subjected to cytokine profiling using a CytoFLEX S flow cytometer (Beckman Coulter, Indianapolis, IN, USA) with fluorescence-encoded beads (LEGENDplex, BioLegend, San Diego, CA, USA). We utilized the mouse inflammation panel (13-plex for IL-1α, IL-1β, IL-6, IL-10, IL-12p70, IL-23, IL-27, IFN-β, IL-17A, MCP-1, TNF-α, IFN-γ, GM-CSF) were selected. All cytokines were detected, except IL-10 and IL-12p70 (Supplementary Figure 5).
